# Key Modulators of the Stress Granule Response TIA1, TDP-43, and G3BP1 Are Altered by Polyglutamine-Expanded ATXN7

**DOI:** 10.1007/s12035-022-02888-2

**Published:** 2022-06-10

**Authors:** Frida Niss, Laura Piñero-Paez, Wajiha Zaidi, Einar Hallberg, Anna-Lena Ström

**Affiliations:** 1 Department of Biochemistry and Biophysics, Stockholm University, Stockholm, Sweden; 2 Present Address: Science for Life Laboratory, Department of Women’s and Children’s Health, Karolinska Institutet, Solna, Sweden; 3 Present Address: Department of Biomedical and Clinical Sciences, Division of Neurobiology, Linköping University, Linköping, Sweden

**Keywords:** Neurodegeneration, Spinocerebellar ataxia type 7, TDP-43, G3BP1, TIA1, Stress granules

## Abstract

Spinocerebellar ataxia type 7 (SCA7) and other polyglutamine (polyQ) diseases are caused by expansions of polyQ repeats in disease-specific proteins. Aggregation of the polyQ proteins resulting in various forms of cellular stress, that could induce the stress granule (SG) response, is believed to be a common pathological mechanism in these disorders. SGs can contribute to cell survival but have also been suggested to exacerbate disease pathology by seeding protein aggregation. In this study, we show that two SG-related proteins, TDP-43 and TIA1, are sequestered into the aggregates formed by polyQ-expanded ATXN7 in SCA7 cells. Interestingly, mutant ATXN7 also localises to induced SGs, and this association altered the shape of the SGs. In spite of this, neither the ability to induce nor to disassemble SGs, in response to arsenite stress induction or relief, was affected in SCA7 cells. Moreover, we could not observe any change in the number of ATXN7 aggregates per cell following SG induction, although a small, non-significant, increase in total aggregated ATXN7 material could be detected using filter trap. However, mutant ATXN7 expression in itself increased the speckling of the SG-nucleating protein G3BP1 and the SG response. Taken together, our results indicate that the SG response is induced, and although some key modulators of SGs show altered behaviour, the dynamics of SGs appear normal in the presence of mutant ATXN7.

**Supplementary Information:**

The online version contains supplementary material available at 10.1007/s12035-022-02888-2.

## Introduction

Spinocerebellar ataxia type 7 (SCA7) is a lethal neurodegenerative disease, which is part of a group of disorders termed polyglutamine (polyQ) diseases. The disorder is caused by an autosomal dominant mutation which results in the expansion of a glutamine repeat in the N-terminal of the ataxin-7 (ATXN7) protein. Although ATXN7 is ubiquitously expressed, a subset of neurons in the central nervous system is especially affected [1], resulting in ataxia and blindness in the patient. ATXN7 normally functions within the deubiquitination (DUB) module of the STAGA/TFTC complex and participates in transcriptional regulation [2, 3]. Mutated ATXN7 can still incorporate into the STAGA/TFTC complex, but this interferes with the normal transcriptional function of the complex [4, 5]. In addition, STAGA/TFTC function is also disrupted by sequestration of the DUB module into protein aggregates formed by mutant ATXN7 [6, 7]. Moreover, apart from the normal interaction partners of ATXN7, a whole host of other proteins can also be found in the aggregates, all of whose loss likely could exacerbate the pathology [for review, see 8]. Interestingly, a recent study using mass spectrometry to investigate the protein content of aggregates in a different polyglutamine disease, Huntington’s disease (HD), found that an unexpectedly high proportion of the aggregate was made up of RNA binding proteins (RBPs) [9]. Furthermore, the RBPs that were sequestered into aggregates disproportionately often contained prion-like domains (PrLDs), indicating that the expanded polyglutamine domain of Huntingtin, the protein mutated in HD, readily interacts with PrLD containing proteins and sequesters them into aggregates. In line with this, in SCA7 disease models and patients, several RBPs have also been found to localise to ATXN7 aggregates [10, 11].

As RBPs have many RNA and protein interaction partners and regulate RNA processing at several different steps [12], numerous important functions could be disturbed by interference of RBPs. In fact, mutations in several RBPs, including FUS and TDP-43, can cause neurodegenerative diseases [13–15]. The causative mutations in these two specific RBPs are often found in the C-terminus that controls the nuclear/cytoplasmic shuttling of the protein, resulting in cytoplasmic accumulation of the respective RBP. This indicates that mutations of FUS and TDP-43 could cause a loss-of-function effect in the nucleus or a gain-of-function effect in the cytoplasm. It has been observed that mislocalisation of FUS and TDP-43 to the cytoplasm dysregulates some of their RNA targets [11, 16] and also can result in pathological cytoplasmic granule assemblies [17]. Thus, mutations that cause mislocalisation of these RBPs seem to result in tandem loss-of-function and gain-of-function effects. Since these effects are evidently pathogenic enough to bring on cell death and neurodegeneration in other neurodegenerative diseases, we hypothesised that sequestration-mediated dysregulation of RBPs should be able to exacerbate SCA7 pathology. Indeed, in a previous study, we could show that transcripts normally controlled by the RBP FUS were dysregulated in a stable inducible SCA7 cell model expressing expanded ATXN7 [11]. Furthermore, since FUS is also involved in DNA repair [18], we were not surprised to find that there was an induction of DNA damage in mutant ATXN7 expressing cells as well. This contributed to the already ascertained list of various negative effects produced by expanded ATXN7 in cells, such as oxidative stress and disruptions of the proteasome and autophagy systems [19–21]. To cope with these types of stresses, cells can induce a specific type of response called stress granules (SGs), which are cytoplasmic, membraneless organelles formed by RBPs and stalled polysomes through a controlled oligomerisation process [22]. These organelles are believed to increase cell survival by sorting mRNAs, allowing translation of necessary transcripts, while inhibiting translation of unnecessary RNAs, as well as regulating various signalling pathways and apoptosis during stress [23]. Following stress relief, the SGs should rapidly disassemble, restoring translation of all RNA transcripts. However, formation of SGs has also been suggested to seed the formation of pathological protein aggregates in several neurodegenerative diseases and thereby worsen pathology [23].

In light of the many mentioned similarities between neurodegenerative diseases when it comes to molecular stress pathology, we were eager to investigate the role of SGs in SCA7, a disease in which SGs have not been studied specifically. We could show that mutant ATXN7 induces the SG response and that two SG-related RBPs, TDP-43 and TIA1, are sequestered into mutant ATXN7 aggregates. Interestingly, mutant ATXN7 was also shown to co-localise with and affect the shape of the induced SGs. Despite this, SG assembly and disassembly in response to arsenite treatment was not affected in SCA7 cells. Moreover, this co-localisation did not result in an increase of the number of ATXN7 aggregates per cell, although a small, non-significant increase of mutant ATXN7 aggregated material could be detected.

## Results

### TDP-43 Co-localises with Cytoplasmic ATXN7 Aggregates

**Fig. 1:**
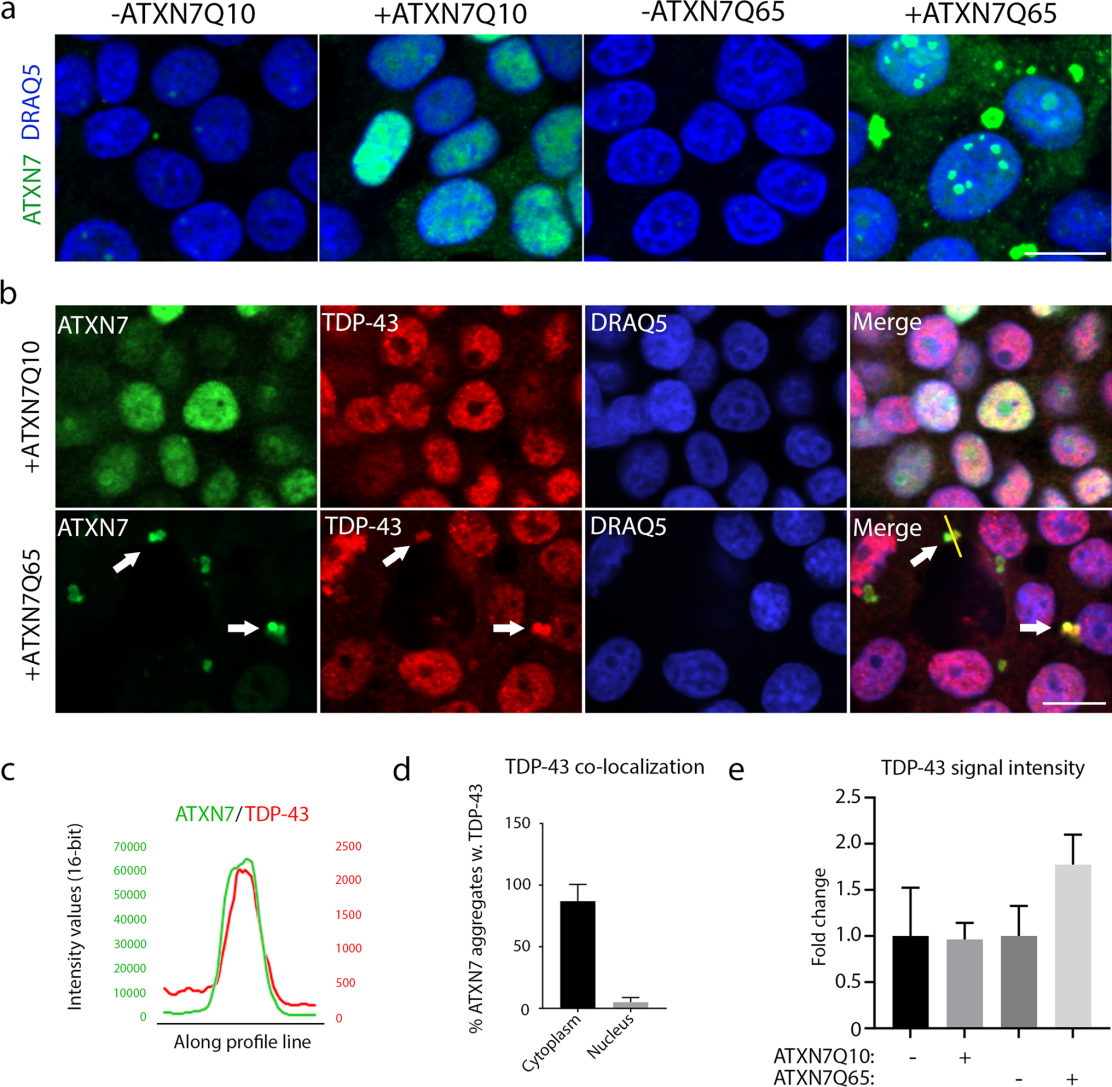
RNA binding protein TDP-43 co-localises with ATXN7 aggregates. **a** Representative confocal images showing PC12 cells induced to express either ATXN7Q10 or ATXN7Q65, as well as non-induced controls. All are stained with ATXN7 in green and DRAQ5 (blue) used as a DNA marker. Contrast settings are the same for all images, w ithin each stain. **b** ATXN7Q10/Q65 expressing cells stained for ATXN7 (green) and TDP-43 (red), using DRAQ5 as a DNA marker. Arrows point to ATXN7 aggregates and co-localising TDP-43 staining. Contrast settings are the same for all images, within each stain. **c** Representative profile plots showing the intensity values of ATXN7 and TDP-43 along the yellow line drawn in **b** (merge). **d** The percentages of sampled ATXN7 aggregates in cytoplasm and nucleus that contain a co-localising intensity peak of TDP-43 such as that displayed in **c**. **e** Quantified fold change of total TDP-43 intensity per cell in ATXN7Q10/Q65 expressing or non-induced cells. *n* = 3 for Q10, and *n* = 4 for Q65. Data are shown as mean ± SEM. Scale bar represents 10 µm

To investigate if stress granules (SGs), that are nucleated by PrLD containing RBPs [24, 25], are affected by the expression of expanded ATXN7, we used an inducible PC12 cell model expressing human full-length ATXN7 with either 10 (ATXN7Q10) or 65 (ATXN7Q65) glutamines to simulate SCA7 pathology. Both constructs were fused to GFP in the C-terminal. After 12 days of induction, the ATXN7Q65 cells show advanced cytoplasmic and nuclear aggregate formation (Fig. 1a), as well as accumulation of both full-length and fragmented ATXN7 (Fig. S1), as characterised in previous papers [19, 20, 26]. In comparison, while ATXN7Q10-induced cells also exhibit fragment accumulation (Fig. S1), they show diffuse nuclear localisation of ATXN7 devoid of aggregation (Fig. 1a).

First, we analysed if TDP-43, a PrLD-containing RBP shown to participate in SG formation [27] and heavily implicated in other neurodegenerative diseases, such as amyotrophic lateral sclerosis (ALS) [28, 29] and Alzheimer’s disease (AD) [30, 31], was affected by mutant ATXN7. Through immunofluorescence we could observe a high co-localisation of TDP-43 with ATXN7 aggregates in the cytoplasm, though only 5% of nuclear ATXN7 aggregates contained TDP-43 (Fig. 1b–d). Our images also indicated a slight non-significant increase of total TDP-43 intensity after induction of ATXN7Q65 (Fig. 1e). It has previously been noted that ATXN7Q10 and ATXN7Q65 is cleaved after induction in our cell model, and while the non-cleaved protein remains mostly nuclear, the shorter polyQ tract containing N-terminal fragments is mainly found in the cytoplasm [26]. Based on the higher co-localisation with cytoplasmic aggregates, we therefore suspect that the PrLD of TDP-43 is more prone to interact with the exposed polyQ domain in N-terminal fragments of expanded ATXN7 than the full-length protein.

### TDP-43 Is Hyperphosphorylated in ATXN7Q65-Induced Cells

**Fig. 2:**
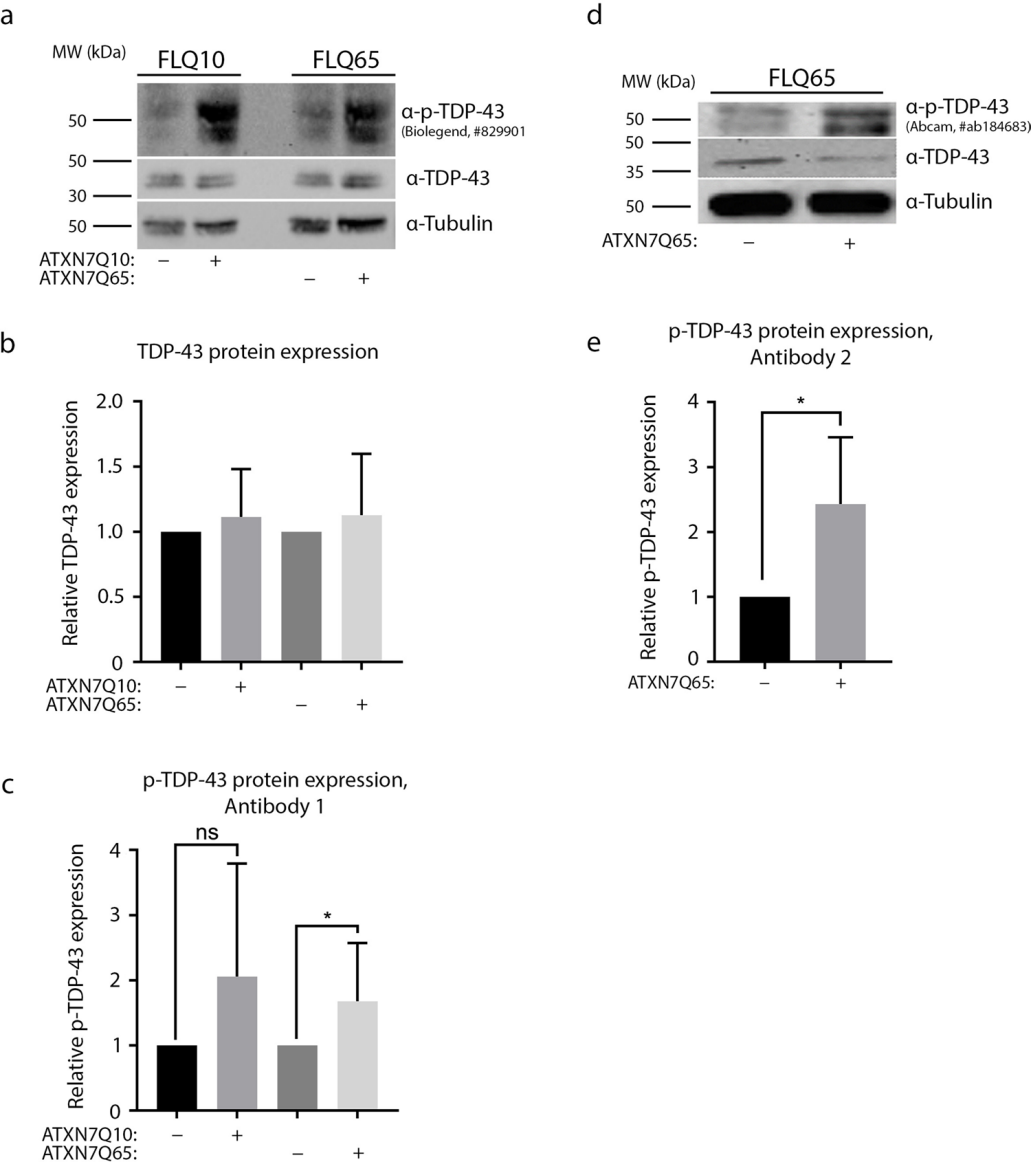
Phosphorylated TDP-43, but not total TDP-43, is increased in ATXN7Q65 expressing cells. **a** Representative western blots probed with α-p-TDP-43 (BioLegend, #829,901), α-TDP-43, and α-Tubulin in ATXN7Q10/Q65-induced or non-induced PC12 cells. **b** Quantified fold change of TDP-43 protein expression from blots shown in **a**. **c** Quantified fold change of p-TDP-43 protein expression from blots shown in **a** (**a**–**c**: *n* = 9). **d** Representative western blots probed with alternative α-p-TDP-43 antibody (Abcam, #184,683), α-TDP-43, and α-Tubulin in ATXN7Q10/Q65-induced or non-induced PC12 cells. **e** Quantified fold change of p-TDP-43 protein expression from blots shown in **d** (**d**–**e**: *n* = 4). Data are shown as mean ± SEM, *p < 0.05

Hyperphosphorylated TDP-43 has previously been observed in cell culture during polyglutamine expression [32], as well as in several other neurodegenerative diseases [33, 34], and has been identified as a pathological marker [35]. Moreover, phosphorylated TDP-43 has previously been shown to localise to ATXN7 aggregates in SCA7 patients [36]. Therefore, we wanted to investigate if TDP-43 hyperphosphorylation occurred in our SCA7 cell model as well. Using western blots, we analysed the expression of TDP-43 after induction of ATXN7Q10 and ATXN7Q65 (Fig. 2). While there was no change in overall TDP-43 protein expression (Fig. 2b), we were in fact able to observe a significant increase in phosphorylated TDP-43 at serine 409/410, using two separate antibodies, when ATXN7Q65 was induced (Fig. 2c and e). We observed a similar trend in cells induced to express ATXN7Q10, though the variation between samples was too high for any conclusions to be drawn.

### TIA1 Co-localises with ATXN7 Aggregates, While G3BP1 Does Not

**Fig. 3:**
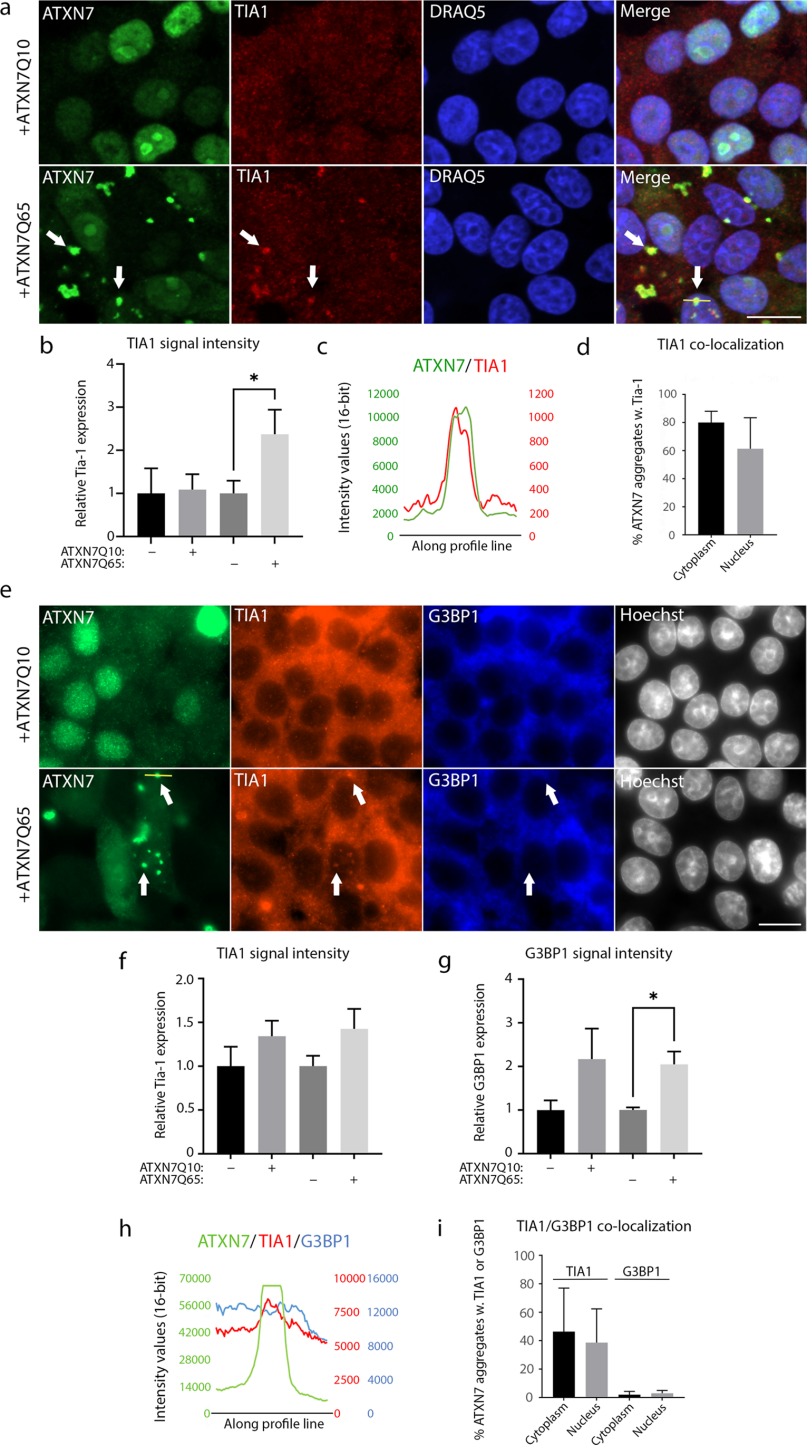
TIA1 is sequestered into aggregates and is increased in ATXN7Q65 expressing cells. **a** Representative confocal images of ATXN7Q10/Q65 expressing cells stained for ATXN7 (green) and TIA1 (red), using DRAQ5 as a DNA stain. Arrows point to ATXN7 aggregates and co-localising TIA1 staining. Contrast settings are the same for all images, within each stain. **b** Quantified fold change of total TIA1 intensity per cell in ATXN7Q10/Q65 expressing or non-induced cells. **c** Representative profile plots showing the intensity values of ATXN7, TIA1, and DRAQ5 along the yellow line drawn in a (merge). **d** The percentages of sampled ATXN7 aggregates in cytoplasm and nucleus that contain a co-localising intensity peak of TIA1 such as that displayed in **c** (**a**–**d**: *n* = 2 for Q10, *n* = 3 for Q65). **e** Representative widefield images of ATXN7Q10/Q65 expressing cells stained for ATXN7 (green), TIA1 (red), and G3BP1 (blue), using Hoechst as a DNA stain. Arrows point to ATXN7 aggregates and co-localising TIA1 staining. Contrast settings are the same for all images, within each stain. **f** Quantified fold change of total TIA1 intensity per cell in ATXN7Q10/Q65 expressing or non-induced cells. **g** Quantified fold change of total G3BP1 intensity per cell in ATXN7Q10/Q65 expressing or non-induced cells. **h** Representative profile plots showing the intensity values of ATXN7, TIA1, and G3BP1 along the yellow line drawn in **e** (ATXN7 panel). **i** The percentages of sampled ATXN7 aggregates in cytoplasm and nucleus that contain a co-localising intensity peak of TIA1 such as that displayed in **h**, or G3BP1 (**e**–**i**: *n* = 3 for Q10, *n* = 4 for Q65). Data are shown as mean ± SEM, *p < 0.05. Scale bars represent 10 µm

**Fig. 4:**
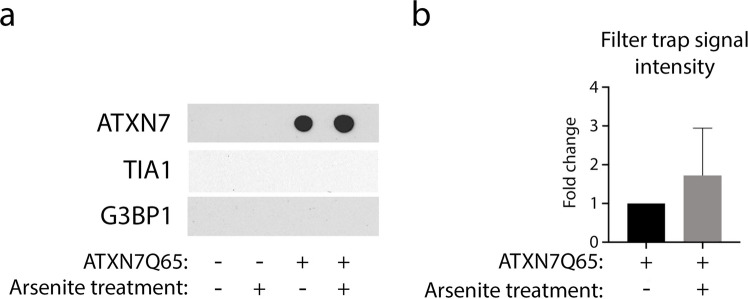
The sequestration of TIA1 into ATXN7Q65 aggregates is not detergent resistant. **a** Representative filter trap blot probed with α-ATXN7, α-TIA1, and α-G3BP1. Insoluble extracts from ATXN7Q65 expressing or non-induced PC12 cells were treated with SDS and DTT before filtration to capture aggregated material and immunoblotting. Contrast settings are the same for all images, within each stain. **b** Quantified fold change of ATXN7 filter trap signal in ATXN7Q65 expressing cells treated with arsenite for 1 h compared to untreated induced cells (*n* = 7). Data are shown as mean ± SEM

TDP-43 can regulate the expression of two nucleating stress granule proteins, TIA1 and G3BP1 [27]. Since TDP-43 was hyperphosphorylated and sequestered into ATXN7 aggregates in SCA7 cells, we therefore next analysed the effect of expanded ATXN7 on TIA1 and G3BP1 localisation and expression. While TIA1 contains a PrLD, G3BP1 does not (PLAAC predictor [37]). As such, we expected there to be a higher chance of interaction between polyQ aggregates and TIA1 than with G3BP1. Indeed, when we immunostained TIA1 together with ATXN7 in induced PC12 cells, we saw that the weak TIA1 staining increased and condensed into the ATXN7 aggregates upon ATXN7Q65 induction (Fig. 3a–b). In fact, TIA1 intensity peaks were observed in as many as 80% of cytoplasmic aggregates and 60% of nuclear aggregates (Fig. 3c–d). However, filter trap assays could not capture TIA1 in the solubilised aggregated material (Fig. 4a), suggesting that the interaction between the two proteins could not withstand harsh denaturing conditions. To be able to stain ATXN7, TIA1 and G3BP1 together with a DNA stain, we then used a different primary antibody against TIA1 as well (Fig. 3e). Using this antibody, we could see the same trend of increased TIA1 immunofluorescence in ATXN7Q65-induced cells (Fig. 3f). However, a similar increase could also be observed in ATXN7Q10-induced cells. Similarly, an increase in G3BP1 could also be observed in both induced ATXN7Q10 and ATXN7Q65 cells, though the increase was only significant in cells induced to express the mutant protein (Fig. 3g). As expected, when we analysed the ATXN7Q65 aggregates for TIA1 and G3BP1 co-localisation, we observed that while it was common for TIA1 to co-localise with the aggregates in both the cytoplasm and nucleus, there was virtually no co-localisation of G3BP1 with the aggregates (Fig. 3h–i). Neither could we find G3BP1 in the filter trap of solubilised aggregated material from ATXN7Q65-induced cells (Fig. 4a).

### G3BP1 Exhibits Speckling Behaviour in ATXN7Q65-Induced Cells

**Fig. 5:**
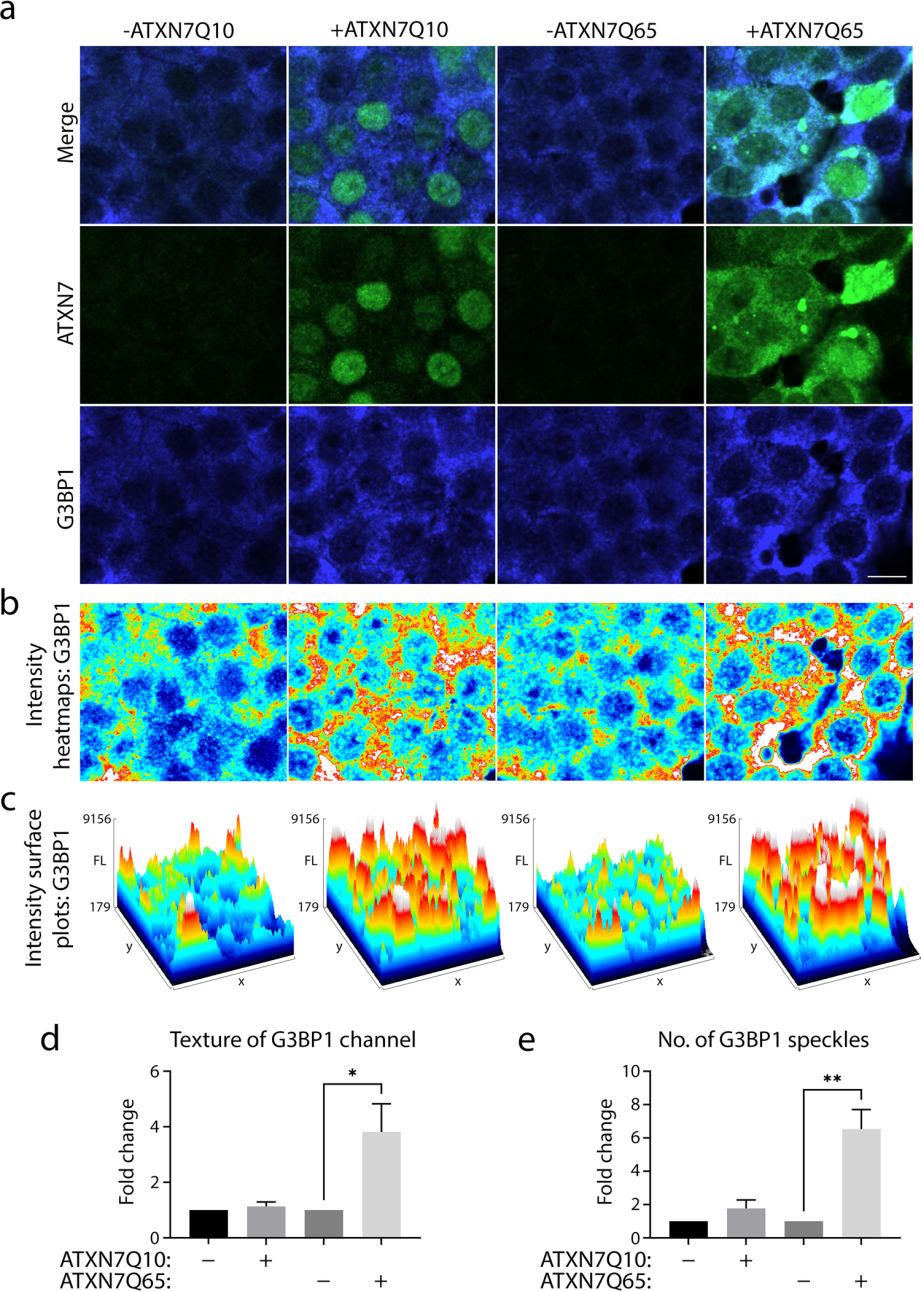
G3BP1 distribution exhibits more texture and a speckling behaviour in ATXN7Q65 expressing cells. **a** Representative confocal images of ATXN7Q10/Q65 expressing or non-induced cells stained for ATXN7 (green) and G3BP1 (blue). **b** Intensity heat maps of the respective G3BP1 stainings shown in **a**. **c** Intensity surface plots of the respective G3BP1 stainings shown in **a**. **d** Quantified fold change of the texture of the G3BP1 IF signal in ATXN7Q10/Q65 expressing or non-induced cells. **e** Quantified fold change of the number of G3BP1 speckles in ATXN7Q10/Q65 expressing or non-induced cells (*n* = 4); data are shown as mean ± SEM, *p < 0.05, **p < 0.01. Scale bar represents 10 µm

When staining for the various stress granule proteins, we noticed that although G3BP1 did not co-localise with ATXN7Q65 aggregates, the G3BP1 signal appeared different in the mutant cells. Using heat maps and surface plots, we could visualise this difference and show that the G3BP1 signal was more granulated in ATXN7Q65-induced cells, compared to non-induced cells or ATXN7Q10-induced cells (Fig. 5a–c). ATXN7Q10-induced cells did show higher intensity peaks than non-induced cells as well though. Therefore, we wanted to further analyse and better quantify the G3BP1 behaviour. For this we used a CellProfiler pipeline to assay variance, determining the texture of the signal. Indeed, we found a fourfold increase in texture of the G3BP1 signal in ATXN7Q65-induced cells compared to non-induced controls and ATXN7Q10-induced cells (Fig. 5d). This led us to believe that the stress induced by ATXN7Q65 expression is causing G3BP1 to start exhibiting speckling behaviour, preparing for stress granule formation. In fact, when quantifying the few stress granule like speckles that were formed by G3BP1 in our non-induced or induced cells, we found an approximate sixfold increase of speckles in ATXN7Q65-induced cells compared to non-induced cells (Fig. 5e), though no such striking difference was found in ATXN7Q10-induced cells.

### Stress Granule Formation Is Enhanced by Expanded ATXN7

**Fig. 6:**
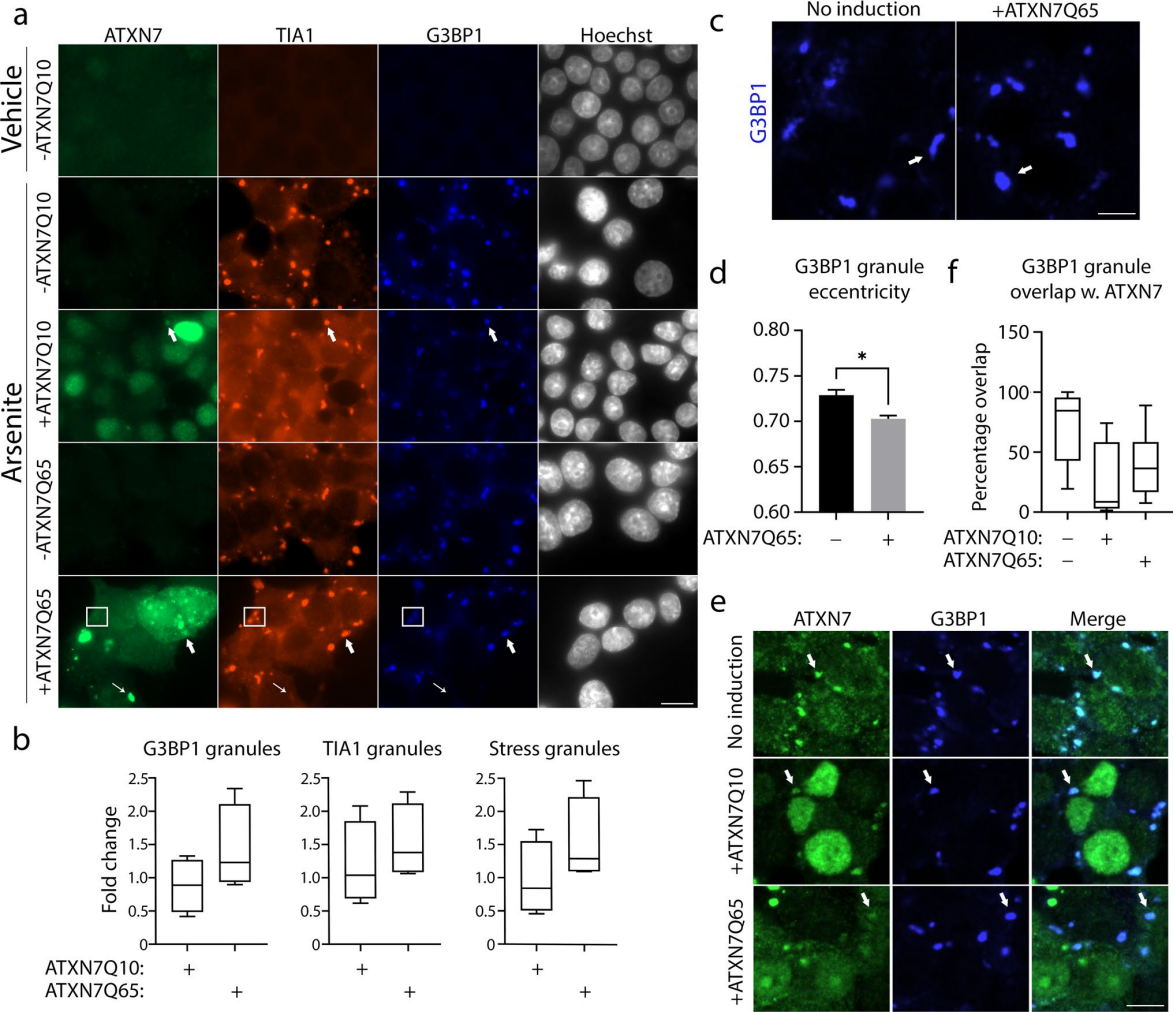
G3BP1 granules are induced and altered in ATXN7Q65 cells. **a** Representative widefield images of ATXN7Q10/Q65 expressing or non-induced PC12 cells treated with arsenite and stained for ATXN7 (green), TIA1 (red), and G3BP1 (blue), using Hoechst as a DNA stain. Thick arrows point to stress granules containing ATXN7, boxes surround stress granules that do not contain ATXN7, and thin arrows point to ATXN7 aggregates containing TIA1. Contrast settings are the same across all images, within each stain. Scale bar represents 10 µm. **b** Quantified fold change of the number of G3BP1 granules, TIA1 granules, and stress granules (stained with both G3BP1 and TIA1) in induced PC12 cells compared to non-induced PC12 cells (*n* = 4). **c** Representative confocal images of G3BP1 granules in arsenite-treated ATXN7Q65-induced PC12 cells compared to non-induced cells. Scale bar represents 5 µm. **d** Quantified eccentricity of G3BP1 granules in arsenite-treated ATXN7Q65-induced PC12 cells compared to non-induced cells (**c**–**d**: *n* = 3). **e** Representative confocal images of arsenite-treated ATXN7Q10/Q65 expressing or non-induced PC12 cells showing co-localisation of ATXN7 with G3BP1 granules. Contrast settings are the same for all images, within each stain. Scale bar represents 5 µm. **f** Percentage of G3BP1 granules positive for ATXN7 in ATXN7Q10/Q65 expressing or non-induced cells (**e**–**f**: *n* = 3). Data are shown as mean ± SEM, *p < 0.05

**Fig. 7:**
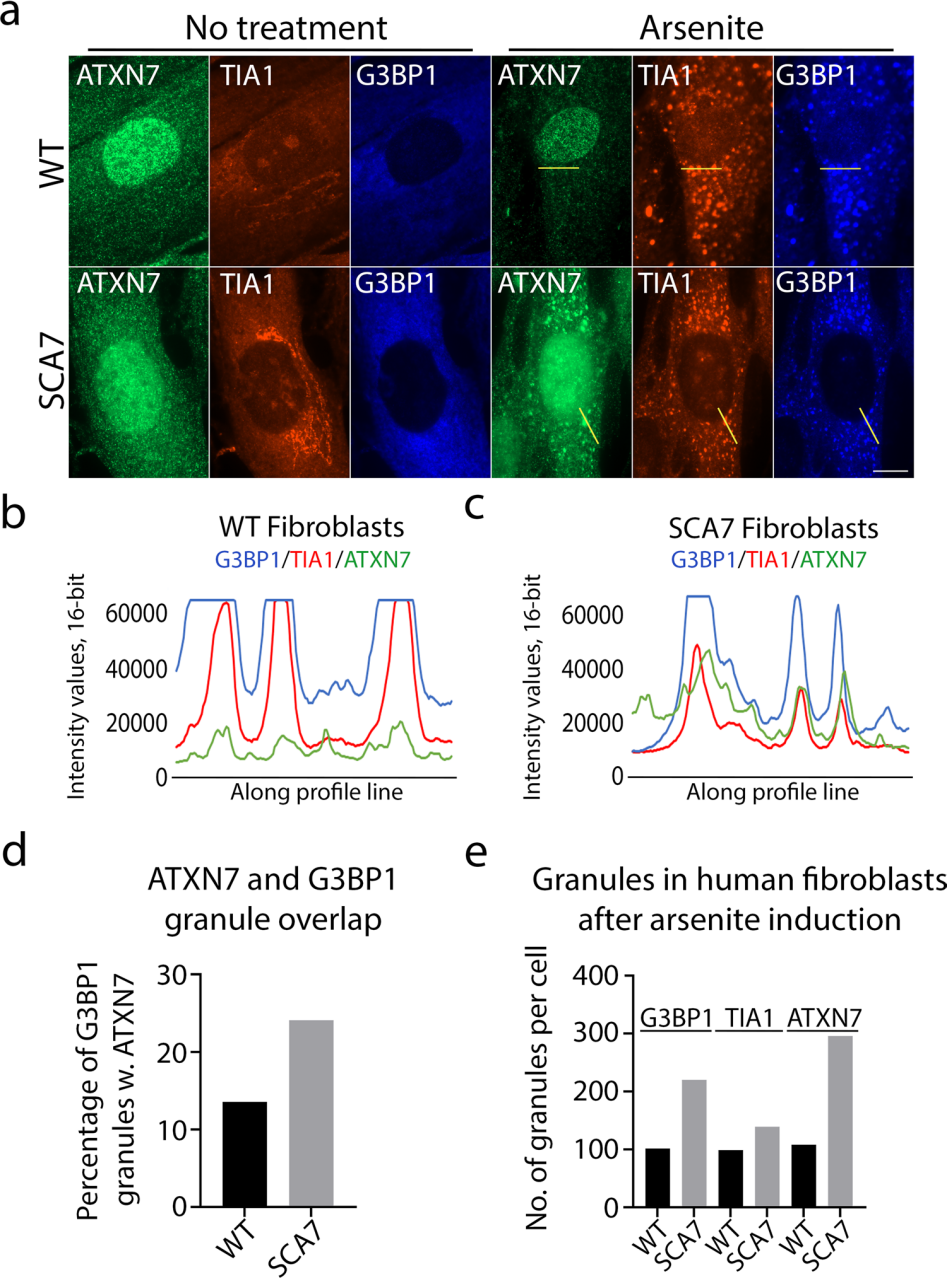
SCA7 patient fibroblasts show co-localisation of expanded ATXN7 with stress granules. **a** Representative widefield images of human control fibroblasts (WT) compared to SCA7 patient fibroblasts (SCA7) stained for ATXN7 (green), TIA1 (red), and G3BP1 (blue). Contrast settings were optimised for untreated cells and arsenite-treated cells separately. **b** Representative profile plots showing the intensity values of G3BP1, TIA1, and ATXN7 in control fibroblasts, along yellow lines drawn in a. **c** Representative profile plots showing the intensity values of G3BP1, TIA1, and ATXN7 in SCA7 fibroblasts, along yellow lines drawn in **a**. **d** Quantified percentage of G3BP1 granules containing ATXN7 staining in human control fibroblasts and SCA7 patient fibroblasts. **e** Quantified number of G3BP1, TIA1, and ATXN7-positive granules in arsenite-treated fibroblasts (*n* = 51 and 54 for WT and SCA7 cells respectively, one replicate). Scale bar represents 10 µm

In light of the effect ATXN7Q65 induction had on TIA1 and G3BP1, two important nucleators of SGs, next we investigated SG formation in our PC12 SCA7 cell model. As G3BP1 did not co-localise with ATXN7Q65 aggregates, we used mainly this protein to assay stress granule formation. To induce robust SG formation, we used arsenite, a compound causing oxidative stress. After 1 h of arsenite treatment, we could ascertain that PC12 cells readily formed G3BP1 and TIA1, as well as double positive (defined as SGs) granules (Fig. 6a–b). ATXN7Q65-induced cells also contained aggregates as usual that were distinguished from the SGs by shape, size, and G3BP1 content. As pointed out in Fig. 6a (thin arrows), we could still see TIA1, but not G3BP1, co-localising with mutant ATXN7Q65 aggregates during arsenite stress conditions. However, TIA1 was also readily observed to co-localise with G3BP1 granules and thus seems to be able to participate in SG formation despite sequestration into ATXN7Q65 aggregates. Thus, the formation of SGs does not appear to be inhibited by ATXN7Q65 expression. In fact, in agreement with the behaviour of G3BP1 in non-arsenite-stressed cells, the stress caused by expanded ATXN7 seems to cause a trend towards more G3BP1 and TIA1 as well as G3BP1-TIA1 double-positive granules (Fig. 6b). The smaller trend towards more granules in ATXN7Q65-induced cells after arsenite treatment, compared to the significantly higher G3BP1 speckling before additional stress application, may be because the upper cellular limit of how many SGs can be formed is approached with arsenite treatment, thus reducing the observable difference between control and mutant cells. To further investigate if mutant ATXN7 results in a stronger SG response, human fibroblasts from a young SCA7 patient, or an apparently healthy control individual of similar age, were investigated. Consistently with our PC12 data, the SCA7 fibroblasts showed an increased number of G3BP1 and TIA1-positive granules compared to the control fibroblasts, upon arsenite treatment (Fig. 7a, e). This is in accord with a study that observed that HD cells responded more robustly with SG formation during ER stress compared to control cells [38]. Taken together, these data indicate that expansion of ATXN7 causes stress that triggers the SG response.

### Expanded ATXN7 Co-localises with Arsenite-Induced G3BP1 Granules and Affects Their Shape

Although we could not observe G3BP1 co-localising to ATXN7 aggregates in non-stressed cells (Fig. 3), a clear co-localisation of mutant ATXN7 to a subset of G3BP1/TIA1 double-positive stress granules could be observed upon arsenite treatment (Fig. 6a, thick arrows). Moreover, throughout imaging of arsenite treated cells, we noticed that the shape of G3BP1 granules was different in non-induced controls compared to in ATXN7Q65-induced cells. While controls exhibited more elongated G3BP1 granules, ATXN7Q65-induced cells showed more rounded granules (Fig. 6c). This was confirmed by measuring the eccentricity (Fig. 6d), as well as the form factor (Fig. S2) of the G3BP1 granules in CellProfiler. Neither the area nor the perimeter was significantly different in the two granule populations (Fig. S2).

Surprisingly, when imaging using a confocal microscope to capture the weaker signal from the endogenous rat ATXN7, we found that endogenous rat ATXN7 also could co-localise with arsenite-induced G3BP1 granules (Fig. 6e). When quantifying the co-localisation of the G3BP1-positive granules with ATXN7-positive granules through immunofluorescence, there was a clear overlap of the two signals after treatment with arsenite, both with endogenous rat ATXN7 and overexpressed human ATXN7Q10/Q65 (Fig. 6f). Consistent with this, we could detect the occurrence of human endogenous ATXN7 in SGs in SH-SY5Y cells and control human fibroblasts, although the association was weaker in these cells (Fig. S3 and Fig. 7a–d). In contrast, a stronger overlap between ATXN7 and G3BP1/TIA1 could be observed in human SCA7 fibroblasts (Fig. 7a–d). SCA7 patient fibroblasts also exhibit a clear condensation of ATXN7 into speckles upon arsenite addition, compared to control human fibroblasts (Fig. 7a, e). Taken together, these data indicate that wild-type ATXN7 may interact with SGs and that this interaction might become stronger upon repeat expansion.

### Expanded ATXN7 Does Not Interfere with Stress Granule Recovery

**Fig. 8:**
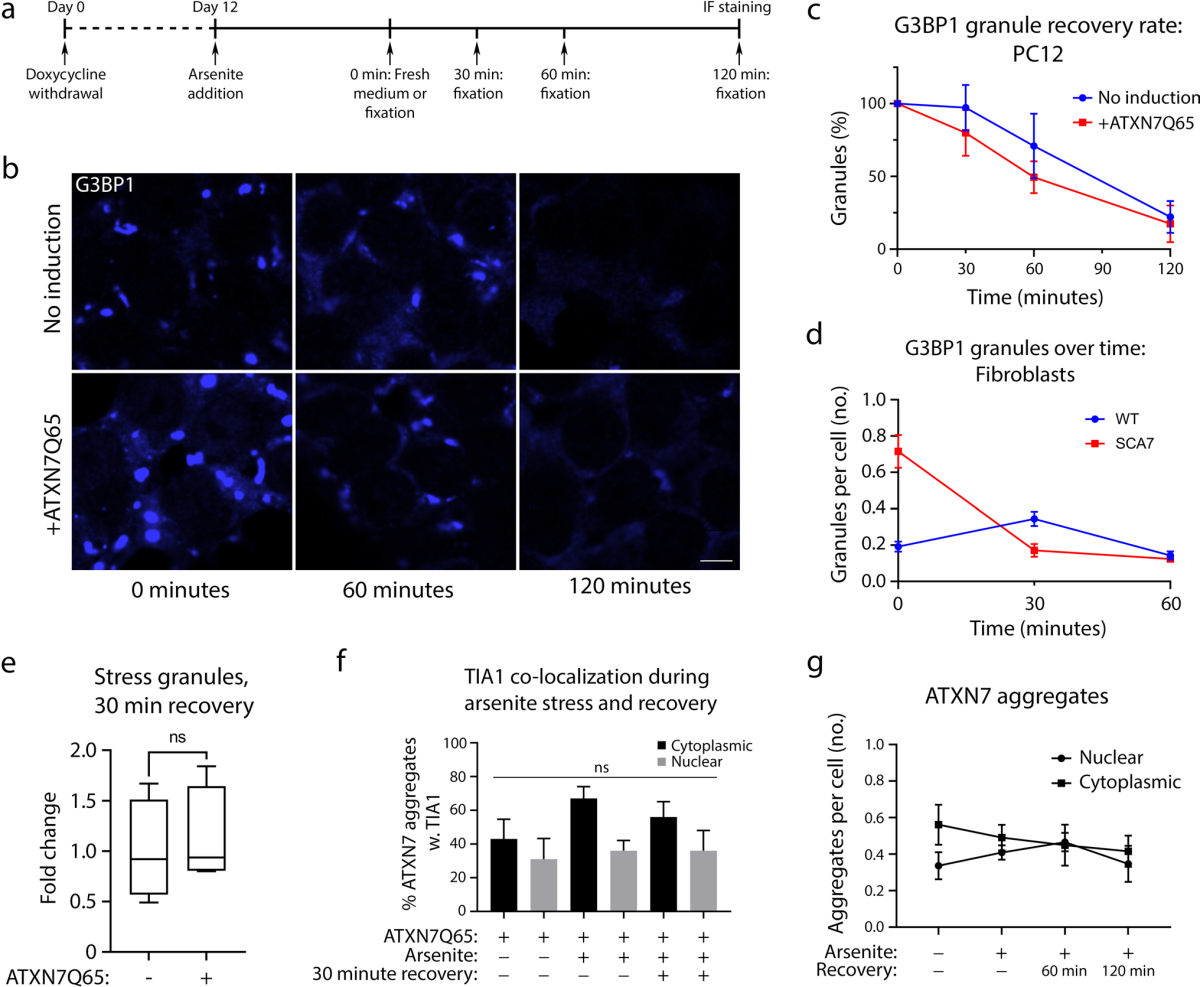
ATXN7Q65 expression does not affect the stress granule recovery rate after arsenite treatment. **a** Schematic explaining the timeline of the experiment. **b** Representative confocal images of ATXN7Q65 expressing or non-induced PC12 cells stained for G3BP1 after the treatment with and recovery from arsenite. **c** Recovery rate of G3BP1 granules in PC12 cells over time, normalised to the number of granules at time point 0 (no recovery) (*n* = 4). **d** Recovery rate of G3BP1 granules after arsenite treatment over time in fibroblasts (*n* = 55–88 cells per line and time-point, one replicate). **e** Recovery of stress granules (TIA1/G3BP1 double-positive speckles) 30 min after removal of arsenite treatment in ATXN7Q65 expressing or non-induced cells, represented in fold change of non-induced cells (*n* = 4). **f** Percentage of ATXN7 aggregates that are TIA1 positive before during and after arsenite stress (*n* = 4). **g** Number of nuclear and cytoplasmic ATXN7 aggregates before, during, and after arsenite treatment (*n* = 4). Data are shown as mean ± SEM; ns, non-significant. Scale bar represents 5 µm

Upon stress alleviation, SGs should rapidly disassemble. However, the presence of aggregating, pathological proteins in SGs have been proposed to potentially prevent this process [23]. We were therefore curious to see if SG clearance following recovery from stress, i.e. arsenite treatment, was affected by ATXN7Q65. To analyse this, induced or non-induced PC12 cells were treated with arsenite for 1 h, allowed to recover from the treatment for 0–120 min, and the number of G3BP1-positive granules determined at different time points (Fig. 8a–b). Surprisingly, no difference in the SG disassembly rate could be observed between ATXN7Q65-induced cells and non-induced control cells (Fig. 8c). Similarly, while SCA7 patient fibroblasts had a threefold higher SG induction than control fibroblasts, after 1 h of recovery, levels were approximately the same for both cell lines (Fig. 8d). When we repeated the experiment in PC12 cells, with inclusion of TIA1 staining along with G3BP1 staining, we received the same results (Fig. 8e).

Association with SGs have been speculated to seed polyglutamine protein aggregation [23]. However, we could not see any fusion between SGs and aggregates during our immunofluorescence experiments (data not shown). Furthermore, if TIA1 containing SGs fused with ATXN7 aggregates, the number of TIA1-positive ATXN7 aggregates should increase. However, the number of TIA1-positive ATXN7 aggregates remained unaltered in response to SG induction or recovery (Fig. 8f). In addition, quantification of the number of nuclear and cytoplasmic ATXN7 aggregates per cell did not reveal any change upon induction of SGs nor after 2 h of recovery from the arsenite stress (Fig. 8g), although the amount of insoluble ATXN7 captured by filter trap assay did show a small, non-significant trend towards more aggregated ATXN7 upon SG induction with arsenite (Fig. 4b).

Taken together, we conclude that although expanded ATXN7 can localise to SGs, this does not affect the clearance rate of stress granules or have any major impact on seeding new ATXN7 aggregates.

## Discussion

Stress granules represent a conserved component of the cellular response to stress and are crucial for cell survival [23]. In the current study, we have shown that expanded ATXN7 affects the expression and/or behaviour of TDP-43, TIA1, and G3BP1, three RNA-binding proteins involved in the stress granule response. We could show that phosphorylation of S409/S410 in TDP-43 is increased and that TDP-43 co-localises with ATXN7 aggregates in SCA7 cells. This is consistent with previous studies identifying increased TDP-43 phosphorylation and sequestration in SCA7 mice and patients, as well as in several other neurodegenerative diseases [15, 16, 32–34, 36]. Findings in other diseases have indicated that TDP-43 exits the nucleus during stress [27, 39] and that TDP-43 phosphorylation exacerbates TDP-43-related neuropathology [35, 40]. However, while Coudert et al. found that TDP-43 phosphorylation was secondary to aggregation, Hasegawa et al. observed that TDP-43 is more prone to multimerise into filaments after phosphorylation by casein kinase 1 [32, 33]. Interestingly, we found a higher co-localisation of TDP-43 with cytoplasmic, rather than nuclear, ATXN7 aggregates. Therefore, we hypothesise that the stress caused by expanded ATXN7 in our SCA7 cell model could cause TDP-43 to exit the nucleus, interact with cytoplasmic polyQ fragments, and become phosphorylated, which strengthens the interaction and causes accumulation of TDP-43 into aggregates.

Besides TDP-43, we could also observe a clear co-localisation of TIA1 with both nuclear and cytoplasmic ATXN7 aggregates. This together with the fact that TDP-43 has been shown to positively regulate the expression of G3BP1 [27, 41] initially led us to expect decreased soluble levels of TDP-43, TIA1, and G3BP1 in our SCA7 cell model. However, western blot and IF analyses instead revealed similar or statistically significantly increased expression level of these RBPs, suggesting that the stress caused by mutant ATXN7 triggers the stress granule response pathway. As a matter of fact, increased expression of stress granule-nucleating proteins has previously been observed in Huntington’s disease patients and models [42, 43]. Not only were we able to observe an increased level of G3BP1 in our expanded ATXN7 expressing cells, but we also found that the behaviour of G3BP1 changed. Although fully formed G3BP1-positive SGs were rare in the SCA7 cells, G3BP1 displayed a statistically significant increase in signal texture and speckling, indicating condensation of G3BP1 into pre-SG structures in ATXN7 cells. Furthermore, following treatment with the SG inducing compound arsenite, a trend towards more SGs in mutant ATXN7, versus control cells, could also be observed. The fact that the arsenite-induced effect on SG formation in mutant versus control cells was smaller than the observed difference in G3BP1 speckling levels before arsenite treatment is most likely due to the close to maximal induction of the SG response by arsenite, as mentioned previously. Hence, despite the sequestration of TDP-43 and TIA1, the ability of SCA7 cells to induce SGs is not reduced, and in fact mutant ATXN7 appears to induce the SG response.

Although we could not see any sequestration of G3BP1 into mutant ATXN7 aggregates, a clear co-localisation of mutant ATXN7 to SGs could be observed upon arsenite-mediated induction of stress granules in both SCA7 PC12 cells and patient fibroblasts. In fact, polyQ-expanded Huntingtin has been shown to co-localise with G3BP1 and SGs in HD models [38, 42], which together with our data suggests that incorporation of polyQ-expanded proteins could be a common feature in polyQ diseases. The interaction of polyQ-expanded proteins with SGs has further been suggested to promote polyQ aggregation [23]. Although we could observe a small non-statistical increase in total aggregated ATXN7 material using filter trap, we did not detect any increase in the number of ATXN7 aggregates per cell following SG induction, suggesting that SGs do not cause seeding of new ATXN7 aggregates.

Surprisingly, we could also observe that endogenous as well as transgenic, wild-type ATXN7 localised to SGs to some extent. To our knowledge, this is the first time ATXN7 has been linked to SGs, though another STAGA DUB subunit, sus1, has been shown to localise to SGs in yeast [44]. Moreover, the yeast ATXN7 ortholog sgf73, along with the STAGA DUB module, were recently linked to export of essential transcripts in response to stress [45]. Further studies to investigate the potential connection between wild-type ATXN7, the STAGA DUB module, and the stress granule response would thus be interesting.

Despite both endogenous and mutant ATXN7 localising to SGs, we could observe a difference in the shape of the SGs when comparing expanded ATXN7 expressing cells to non-induced control cells, indicating that expanded ATXN7 does not interact with SGs identically to wild-type. An interesting recent paper has compared SG structure after overexpression of several SG proteins, such as TIA1, G3BP1, TDP-43, and FMR1 [46]. They noted that when different SG proteins were overexpressed to induce SG formation, granules of two classes were formed, which they designated as rough and smooth granules. G3BP1 and TDP-43 overexpression resulted in smooth granules, while increased TIA1 levels produced rough granules. While we can speculate that the partial sequestration of TDP-43 and/or TIA1 into polyQ aggregates, as well as the co-localisation of mutant ATXN7 with stress granules could affect granule shape and function, it is important to note that SGs are incredibly complex structures. The nucleation is performed by numerous proteins and mRNAs [47], and the resulting granule is composed of both a stable core and a more dynamic shell [48]. It is therefore difficult to predict how small subtractions or additions of specific proteins will affect shape or function of SGs. From our results, it is clear that arsenite-induced stress granule formation, as well as disassembly following recovery, do not appear to be negatively affected by mutant ATXN7 expression. However, as SG composition can vary depending on the type of stress and cell type, it is still possible that mutant ATXN7 could negatively impact SGs under other circumstances. Furthermore, considering that SCA7 is a chronic disease, it is plausible that SGs in the long run could be altered resulting in deleterious dysfunctions of the stress response and promotion of ATXN7 aggregation. Especially since long-term accumulation of toxic expanded ATXN7 species [1, 49, 50] in SCA7 patients could presumably with time result in large disruptions to PrLD containing RBPs as well as continuous longterm induction of the SG response. In fact, SGs formed in response to chronic stress have been suggested to promote cell death rather than survival [for review, see: 51].

In conclusion, we find that although the stress granule proteins TIA1 and TDP-43 are sequestered into mutant ATXN7 aggregates, this does not appear to inhibit stress granule formation or disassembly. In contrast, a heightened condensation behaviour of G3BP1 and trend towards more stress granules could be observed in SCA7 cells, suggesting that expansion of ATXN7 induces stress that triggers the SG response. Most interestingly, it seems that ATXN7 localises to SGs and that association of the mutant protein into SGs alters the shape of the SGs. The role of this association, as well as the molecular consequences of an altered SG shape in SCA7 disease, needs to be clarified in further studies.

## Materials and Methods

### Cell Culture

All cells were cultured at 37 °C and 5% CO_2_ in a humidified environment. PC12 cells stably transfected with the inducible Tet-Off system controlling ATXN7 expression [26] were cultured in Dulbecco’s Modified Eagle Medium (Gibco, #41,966–029), supplemented with 10% horse serum (Gibco, #16,050–122), 5% tetracycline-screened fetal bovine serum (FBS) (Thermo Fisher Scientific, #SH30070.03), 1% PEST (Gibco, #15,140–122), 0.1% G418 (Gibco, #11,811,031), 100 µg/ml hygromycin (Life technologies, #10,687,010), and 1 µg/ml doxycycline (Merck, #D9891-5G). For induction of ATXN7 expression, doxycycline was removed from the medium for 12 days. SHSY-5Y cells were cultured in Minimum Essential Medium (MEM) (Gibco, #21,090–022), 10% FBS (Gibco, #10,270–106), 1% non-essential amino acids (NEAA) (Gibco, #11,140,035), 1% L-glutamine (Gibco, #25,030,024), and 1% PEST. Fibroblasts (Coriell Institute, #GM03561 and #GM07492) were cultured in MEM supplemented with 20% FBS, 1% NEAA, 1% L-glutamine, and 1% PEST.

### Immunofluorescence

Prior to staining, cells were grown on no. 1.5 coverslips coated with 0.5% gelatin. After induction and/or treatment with 0.5 mM arsenite for 1 h, the cells were rinsed once in PBS and then fixed with 4% PFA for 45 min in room temperature. This was followed by 3 × 10 min in PBST (0.1% Triton-X100 in PBS), 30 min of blocking (10% FBS in PBST), and incubation over night with primary antibodies in primary antibody solution (1% FBS in PBST). The primary antibodies employed in this study were rabbit α-ATXN7 [1, 52] (1:500), mouse α-TDP-43 (Abcam, #ab104223, 1:1000), mouse α-TIA1 (Santa Cruz, #sc-166247, 1:250), goat α-TIA1 (Abcam, #ab61700. 1:200), and mouse α-G3BP1 (Proteintech, #66,486–1, 1:200). After the primary antibody incubation, the coverslips were washed 3 × 10 min in PBST and then incubated with secondary antibodies (Alexa Fluor 488 α-rabbit/568 α-mouse/568 α-goat/633 α-mouse, Thermo Fisher Scientific, 1:5000) in blocking solution for 1 h. 5 µM DRAQ5 (Thermo Fisher Scientific, #62,251) or 5 µg/ml Hoechst (Sigma-Aldrich) was added as a DNA marker to the secondary antibody solutions. The coverslips were then washed 3 × 10 min in PBST, mounted with Fluoromount-G (Southern Biotech, #0100–01) on microscope slides, and sealed with nail polish.

### Image Acquisition and Analysis

3-channel images were captured using a Zeiss Axiovert 200 multipoint confocal microscope with a 63 × /1.4 NA oil immersion objective, and the Micromanager open-source software [53]. 4-channel images and images of fibroblasts were captured using an Axio Observer ATXN7 widefield microscope with a 100 × /1.4 NA oil immersion objective and the Zen Blue software (Zeiss). Representative images were handled in FIJI open-source software [54], and brightness/contrast settings were constant for each set of representative images, as indicated in the figure legends. This resulted in diffuse or endogenous staining displayed being weak compared to the various aggregate and speckle stainings. For analysis of aggregate co-localisation with TDP-43, TIA1, or G3BP1, the images were scored as follows. Five cytoplasmic and five nuclear aggregates from five randomly selected images in each replicate were assessed. Through each aggregate, a line was drawn using the FIJI software. The intensity profiles of each stain was compared to the ATXN7 channel, and if there was a coinciding intensity peak twofold higher than the immediate background staining, the aggregate was scored as positive for co-localising stain. Heat maps and surface plots showcasing the G3BP1 staining were created using the ‘Lookup Table: royal’ and ‘Surface Plot’ functions in FIJI. The total intensity of each image was measured using the open-source software CellProfiler [55] and normalised to the number of nuclei in the image, counted manually or automatically. Likewise, CellProfiler was employed to assess the number of G3BP1 speckles, TIA1 speckles, ATXN7 speckles, and stress granules after arsenite induction, while the number of G3BP1 speckles in non-stressed cells were counted manually according to the following rules. The speckle must be shaped like an arsenite-induced speckle, i.e. rounded or oval-shaped, and the G3BP1 staining intensity must be eightfold higher compared to immediate background. For automatic speckle counting, the images were thresholded using a global Otsu threshold with three classes assigning the middle class to background. The minimum threshold was set for each replicate according to arsenite-treated non-induced PC12 cells. Additionally, CellProfiler was used to measure texture of the G3BP1 staining from confocal images, as determined using the variance output, as well as shape and size of G3BP1 speckles, as determined using the eccentricity, form factor, area, and perimeter outputs.

### Western Blots

Prior to harvest of protein extracts, the cells were seeded in 10-cm Petri dishes. Following induction for 12 days, cells were rinsed once with ice-cold PBS and then lysed with RIPA buffer containing a protease inhibitor cocktail for 15 min. Cells were then collected using cell scrapers and centrifuged at 4 °C and 20 000 × *g* for 10 min. The pellet was discarded, and the protein concentration of the supernatant was determined using a Bradford assay. 6 × Loading dye containing SDS was added to each sample before they were boiled for 10 min at 95 °C, vortexed and loaded onto a 8% polyacrylamide gel using apparatus from Bio-Rad. After electrophoresis, the samples were blotted onto a 0.2 µm nitrocellulose membrane. The membrane was rinsed with PBS and blocked for 1 h in 10% dry milk powder in TBST (100 mM Tris-buffered saline pH 7.4 and 0.1% Tween-20). Following blocking the membrane was incubated over night at 4 °C with primary antibody in 2% dry milk in TBST, after which it was rinsed 3 × 10 min in TBST, incubated with secondary antibody (1:50 000, HRP-conjugated, Invitrogen) in 2% dry milk in TBST, and rinsed 3 × 10 min with TBST again. The primary antibodies used were rabbit α-ATXN7 [1, 52] (1:700), mouse α-TDP-43 (Abcam, #ab104223, 1:10 000), rat α-TDP-43 (S409/S410) (BioLegend, #829,901, 1:500), rat α-TDP-43 (S409/S410) (Abcam, #ab184683, 1:1000), and mouse α-Tubulin (Sigma, #T9026, 1:1000). The secondary antibody was detected using the SuperSignal™ West Dura Extended Duration Substrate (Thermo Fisher Scientific, #34,075) and a ChemiDoc XRS + (Bio-Rad). Image analysis was performed using the Image Lab software (Bio-Rad).

### Filter Traps

Cells were seeded into 10-cm Petri dishes and induced prior to harvest. On the 12th day of induction, the cells were rinsed with ice-cold PBS, lysed with RIPA buffer supplemented with protease inhibitor cocktail, and scraped into Eppendorf tubes. They were then centrifuged for 10 min at 4 °C and 21 000 × *g.* The supernatant was discarded, and the pellet was washed twice with RIPA buffer before being re-suspended in DNAse I containing buffer for 1 h. The protein concentration of the pellet suspension was determined using a Bradford assay, and the samples were mixed with SDS and DTT (final concentrations 2% and 100 mM, respectively) and boiled for 5 min. A 0.2 µm nitrocellulose membrane was loaded into a Dot-Blot manifold (Bio-Rad) and washed with 0.1% SDS solution before equal amounts of protein were added from each sample. The samples were vacuum-filtered through the membrane followed by two washes of 0.1% SDS solution. The membrane was then immunoblotted as described previously in the ‘Western Blots’ section.

## Supplementary Information

Below is the link to the electronic supplementary material.

Supplementary file1 (PDF 445 KB)

## Data Availability

The data sets generated during and/or analysed during the current study are available from the corresponding author upon reasonable request.
