# Correction: Key Modulators of the Stress Granule Response TIA1, TDP-43, and G3BP1 Are Altered by Polyglutamine-Expanded ATXN7

**DOI:** 10.1007/s12035-026-05806-y

**Published:** 2026-04-17

**Authors:** Frida Niss, Laura Piñero-Paez, Wajiha Zaidi, Einar Hallberg, Anna-Lena Ström

**Affiliations:** 1 Department of Biochemistry and Biophysics, Stockholm University, Stockholm, Sweden; 2 Science for Life Laboratory, Department of Women’s and Children’s Health, Karolinska Institutet, Solna, Sweden; 3 Department of Biomedical and Clinical Sciences, Division of Neurobiology, Linköping University, Linköping, Sweden

**Correction: Molecular Neurobiology (2022) 59:5236-5251**

10.1007/s12035-022-02888-2

In the original version of this article, a mistake was made in the assembly of figure 4A. The figure should show representative blots for ATXN7, TIA1 and G3BP1 probing following filter trap analysis. Four independent experiments were performed and in all cases a strong signal was observed for ATXN7, whereas no signal could be observed for either TIA1 or G3BP1. By mistake the representative image selected to show that there is no signal for G3BP1 was also added as a representative image showing that there is no signal for TIA1. The mistake is visible if looking closely and paying attention to the weak unspecific background in the original figure 4A.
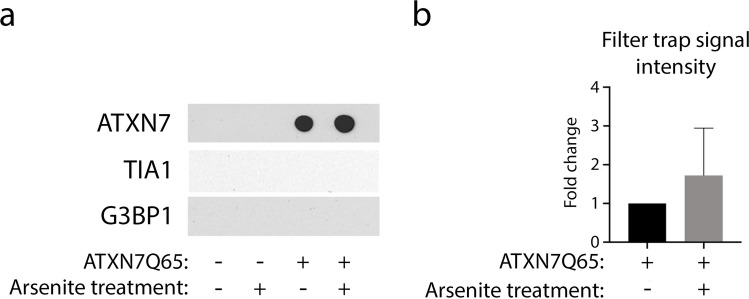


The mistake did not in any way affect how the results should be interpreted.

The original article has been corrected.

