# Supplementary material for: Key Modulators of the Stress Granule Response TIA1, TDP-43, and G3BP1 Are Altered by Polyglutamine-Expanded ATXN7
Source: Mol Neurobiol. 2022 Jun 10;59(8):5236–51. doi: 10.1007/s12035-022-02888-2 (PMC9363381; doi:10.1007/s12035-022-02888-2)
Supplement: Supplementary file 1 — Supplementary file1 (PDF 445 KB) [file 12035_2022_2888_MOESM1_ESM.pdf]

Supplementary Material

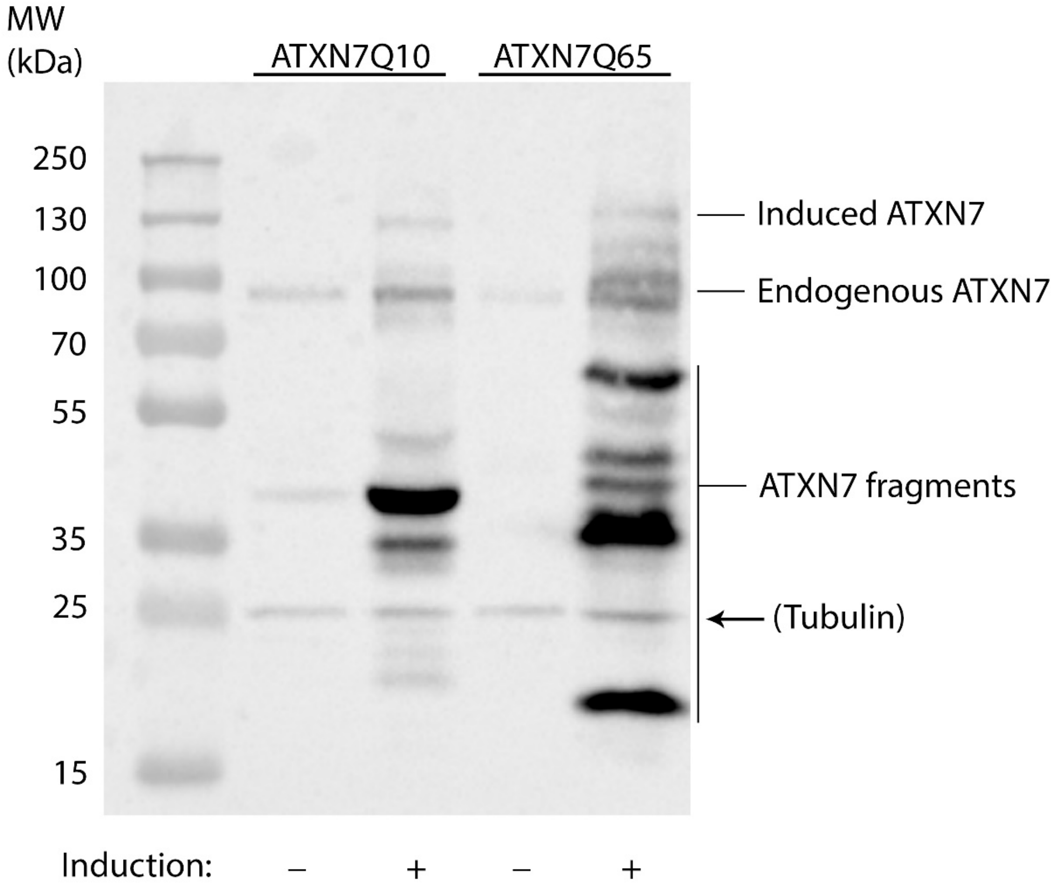

Supplementary Figure S1: ATXN7 induction in inducible PC12 SCA7 cell model. As in previous publications, after 12 days of induction, both ATXN7Q10 and ATXN7Q65 cell lines show strong ATXN7 induction and marked fragment accumulation. Prior to probing for ATXN7, this blot was probed for Tubulin as a loading control for TDP-43.

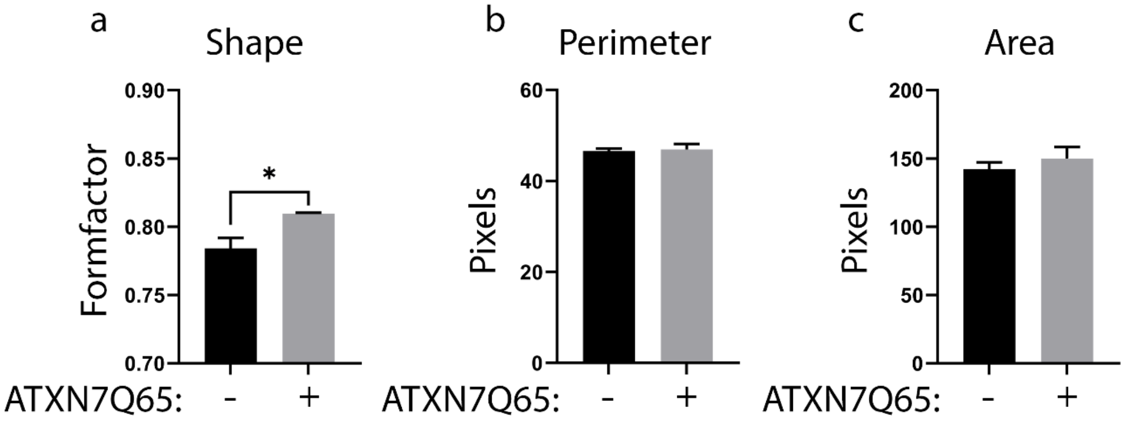

Supplementary Figure S2: a) Formfactor of G3PB1 speckles in ATXN7Q65 induced cells compared to controls. b**)** Perimeter of G3PB1 speckles in ATXN7Q65 induced cells compared to controls. **c)** Area of G3PB1 speckles in ATXN7Q65 induced cells compared to controls. Data are shown as mean ± SEM, *: $p<0.05$.

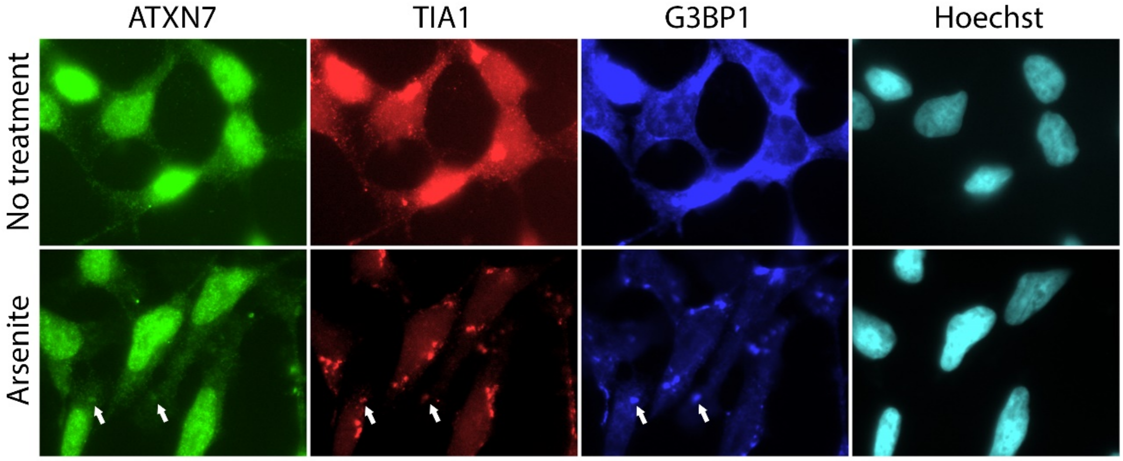

Supplementary Figure S3: Representative widefield images of SHSY-5Y cells stained for ATXN7 (green), TIA1 (red), and G3BP1 (blue), using Hoechst as a DNA stain. Arrows are pointing to stress granules with signal in all three protein channels.
